# Plasma BDNF Is Reduced in Acute Ischemic Stroke Patients With Type 2 Diabetes Mellitus and Associated With Fibrin-Rich Thrombi

**DOI:** 10.1155/jdr/6693363

**Published:** 2025-11-14

**Authors:** Qun Gao, Bo Hei, Shen Hu, Jingru Zhou, Bin Wang, Dongliang Wang, Daming Wang, Jiachun Liu, Jingwen Fan

**Affiliations:** ^1^Department of Neurosurgery, Peking University People's Hospital, Beijing, China; ^2^Department of Neurosurgery, Beijing Hospital, National Center of Gerontology, National Health Commission, Institute of Geriatric Medicine, Chinese Academy of Medical Sciences, Beijing, China; ^3^Department of Laboratory Medicine, Beijing Hospital, National Center of Gerontology, National Health Commission, Institute of Geriatric Medicine, Chinese Academy of Medical Sciences, Beijing, China

**Keywords:** brain-derived neurotrophic factor (BDNF), diabetes mellitus, fibrin, stroke, thrombus

## Abstract

**Aim:**

Acute ischemic stroke (AIS) patients with diabetes mellitus (DM) have significantly lower brain-derived neurotrophic factor (BDNF) levels compared to nondiabetic patients. However, the impact of BDNF on thrombus characteristics is unclear. The relationship between the plasma BDNF and fibrin ultrastructure of thrombi was investigated.

**Methods:**

This study analyzed 72 AIS patients (35 with T2DM) with first-ever stroke and 10 healthy controls. All patients were assessed for severity and type of stroke, risk factors, and levels of plasma BDNF in the acute stroke. Retrieved arterial thrombi from AIS patients underwent histopathological (immunohistochemical, Martius scarlet blue staining, and immunofluorescence), ultrastructural (scanning/transmission electron microscopy), and permeability (CT/CTA) analyses. In vitro effects of recombinant BDNF (rHu-BDNF) on fibrin polymerization (turbidity assay), clot firmness (thromboelastography), and lysis were assessed in plasma pools.

**Results:**

AIS/DM patients exhibited significantly lower plasma BDNF versus AIS/non-DM patients (6.83 ± 0.81 vs. 8.70 ± 0.73 ng/mL, *p* < 0.001) and controls (9.81 ± 0.59 ng/mL). Meanwhile, AIS/DM patients had higher baseline NIHSS scores than AIS/non-DM patients (17.09 ± 8.29 vs. 13.22 ± 6.15, *p* = 0.027). AIS/DM thrombi histopathological showed lower BDNF staining intensity, higher fibrin density, and reduced permeability. Moreover, BDNF levels positively correlated with thrombus permeability (*r* = 0.862, *p* < 0.001). We showed that rHu-BDNF reduced the density of fibrin fiber (*p* < 0.001), turbidity (*p* < 0.05), and maximum clot firmness (*p* < 0.05), while increasing the diameter of fibrin (*p* < 0.05), prolonging thrombin time (*p* < 0.001), and accelerating lysis (*p* < 0.001) in a concentration-dependent manner in plasma from AIS/DM patients.

**Conclusion:**

This study demonstrates that low BDNF levels in AIS/DM patients promote fibrin-rich, dense clot formation, partly via altered fibrin fiber structure and fibrin polymerization. BDNF may serve as a novel biomarker for thrombus heterogeneity and a potential target to improve thrombolysis in diabetic stroke.

## 1. Introduction

Acute ischemic stroke (AIS) stands as the predominant contributor to mortality and long-term disability on a global scale [1]. Type 2 diabetes mellitus (T2DM) affects the occurrence and prognosis of AIS [2]. It is associated with poorer functional outcomes and higher mortality risk [3]. This increased risk is attributed to a complex interplay of hyperglycemia-induced oxidative stress, platelet activation, endothelial dysfunction, and impaired neurovascular coupling.

The brain-derived neurotrophic factor (BDNF) gene, which encodes BDNF, is located on chromosome 11p13, region p13–14 [4]. BDNF is essential for neuronal survival, synaptic plasticity, and neurogenesis, significantly influencing the brain's response to ischemic injury [5, 6]. Research on animals indicates that the hippocampus in the brain is a primary source of BDNF production and secretion [7]. BDNF is expressed in various nonneuronal tissues, with platelets being the primary source of peripheral BDNF [8]. Numerous clinical studies have demonstrated reduced serum BDNF levels in individuals with Type 2 diabetes [9–11]. This may be due to the consumption or downregulation of BDNF in platelets being induced during the progression of diabetes [12]. Patients with AIS exhibit significantly lower serum levels of BDNF compared to healthy individuals, with stroke severity inversely related to BDNF levels [13].

Meanwhile, recent research has highlighted the role of thrombus composition in stroke pathophysiology [14]. Thrombi rich in fibrin are associated with a higher thrombotic burden, resistance to thrombolysis, and an increased risk of poststroke complications [15]. Our previous study showed that thrombi in diabetic patients contained more fibrin and fewer red blood cells (RBCs) compared to those in nondiabetic patients [16]. In AIS patients with T2DM, in addition to the hyperglycemia-induced activation of the coagulation cascade, platelet hyperactivity promotes the formation of dense, fibrin-dominant clots [17]. Furthermore, given that BDNF regulates thrombus stability [18], we hypothesize that BDNF may also be associated with the formation of these fibrin-rich thrombi.

In this study, we aim to elucidate the mechanistic link between the reduction of BDNF and the pathogenesis of fibrin-rich thrombi in patients with AIS and diabetes mellitus (DM) through a comprehensive analysis of thrombus ultrastructure, plasma BDNF levels, and thrombus functional properties such as permeability, polymerization, and lysis. By employing a combination of clinical, histopathological, and in vitro experimental approaches, we seek to determine whether BDNF regulates the properties of fibrin thrombi and clarify its potential role as a novel therapeutic target in improving thrombolytic efficacy and clinical prognosis in diabetic patients with AIS.

## 2. Methods

Consecutive patients diagnosed with AIS and undergoing mechanical thrombectomy (MT) at Peking University People's Hospital and Beijing Hospital from June 2021 to December 2024 were prospectively enrolled in this study. Inclusion criteria included (1) AIS due to an occlusive intracranial clot in either anterior or posterior circulation, (2) availability of preoperative CT data, including NCCT and CTA evaluations, (3) suitable retrieved clots for histopathological and ultrastructural analysis, and (4) blood available for analysis. All study participants provided informed consent. The Beijing Hospital Ethics Committee approved this study as it met national and international guidelines for research on humans. T2DM was confirmed based on the diagnostic criteria for T2DM by the World Health Organization [19]. Further, participants who had a history of T2DM or were currently receiving hypoglycemic therapy were also defined as T2DM patients.

### 2.1. Clinical Data Collection and Assessment

For analytical purposes, we collected data on demographic characteristics (including age and gender), medical history (such as hypertension, dyslipidemia, admission blood glucose level, smoking status, atrial fibrillation, coronary artery disease, and a history of stroke or transient ischemic attack), clinical and laboratory data, use of anticoagulant and/or antiplatelet agents, thrombus location (encompassing the M1 segment of the middle cerebral artery, M2 segment of the middle cerebral artery, anterior cerebral artery [ACA], terminal segment of the internal carotid artery [ICA], and basilar artery [BA]), as well as procedural details. Stroke severity was evaluated using the National Institutes of Health Stroke Scale (NIHSS) score. The etiological classification of stroke was performed in accordance with the criteria from the Trial of ORG 10172 in Acute Stroke Treatment [20].

### 2.2. Plasma Preparation

Blood samples were collected from AIS patients on the day of hospital admission, with controls consisting of 10 healthy individuals undergoing routine physical examinations. Neither the AIS patients nor the control subjects were receiving anticoagulant therapy. Peripheral blood specimens from both groups were divided into two types of vacutainer tubes: those containing EDTA (Becton, Dickinson and Company) for BDNF detection and those with sodium citrate (Greiner, Thailand) for clot and thromboelastographic analyses. These samples were centrifuged at 3000 × *g* for 10 min at 4°C within 15–30 min of collection. The resulting plasma was harvested, aliquoted, and immediately stored at −80°C until subsequent analysis. Plasma BDNF concentrations were measured using the Human BDNF ELISA Kit (intra/intervariability < 10%) (SEKH-0101, Solarbio) following the manufacturer's instructions, with all assays performed by a research assistant blinded to the clinical status of the participants.

### 2.3. Histological Staining

A total of 72 recovered cerebral thrombi were collected; specimens were promptly rinsed with phosphate-buffered saline (PBS) for several minutes, subjected to longitudinal incision, and fixed in either 10% paraformaldehyde or 2% glutaraldehyde before being embedded in paraffin. To assess the overall thrombus structure, 4-*μ*m-thick longitudinal sections were prepared from these samples. Histological analyses included Martius scarlet blue (MSB) staining (G2040, Solarbio, China), immunohistochemical staining for BDNF (ab108319, abcam, United Kingdom), and immunofluorescent staining for fibrin (goat anti-human fibrinogen-fluorescein isothiocyanate, Solarbio, China). Based on MSB staining results, the proportional composition of thrombi (fibrin, RBCs, white blood cells [WBCs], and platelets) was quantified using the Orbit Imaging Analysis machine learning software (http://www.Orbit.bio, Idorsia Ltd.) [21]. For BDNF immunohistochemistry, Image-Pro Plus software (IPP, Version 6.0, Media Cybernetics Corporation, United States) was used to calculate the integral optical density (IOD) per unit area. Confocal microscopy (Olympus Spectral Confocal Microscopy FV1000) was employed to capture images at excitation/emission wavelengths of 488/560 nm. For fibrin immunofluorescence (rabbit polyclonal fibrinogen/FITC, DAKO), immunoreactivity was quantified via IPP software (United States) as the mean gray value intensity—after background subtraction—relative to the total thrombus area, with results expressed in arbitrary fluorescence units (a.u.).

### 2.4. Measurement of Imaging Parameters

As previously described [22], admission imaging utilized a Canon Medical Systems Aquilion ONE CT scanner equipped with a 320 × 0.5 mm detector array. All participants received noncontrast computed tomography (NCCT) followed by CT angiography (CTA). To quantify thrombus permeability, we analyzed attenuation changes within clot regions of interest by comparing NCCT and corresponding CTA datasets. Mean thrombus density was documented in Hounsfield units (HU) for both NCCT (designated CT [HU]) and CTA (designated CTA [HU]). Absolute clot perviousness (*δ*HU) was derived using the formula: *δ*HU = CTA (HU) − CT (HU). Consistent with established criteria [23], thrombi exhibiting *δ*HU ≥ 10 HU were classified as pervious, while those with *δ*HU < 10 HU were considered impervious.

### 2.5. Scanning Electron Microscopy

Seventy-two sample sections cut longitudinally underwent sequential dehydration in an ethanol series, progressing through increasing concentrations (30%, 50%, 75%, 85%, 95%, and 100%) with 10-min incubations at each step. Following dehydration, specimens were subjected to critical point drying using CO_2_, mounted on colloidal carbon stubs, and coated via sputtering. Prepared samples were examined using a JEOL7500 scanning electron microscope (SEM) at Peking University Medical Department. A minimum of six images per specimen were evaluated, with acquisitions performed in randomly chosen regions spanning from sample peripheries to central zones.

### 2.6. Transmission Electron Microscope

Seventy-two specimens designated for transmission electron microscopy (TEM) underwent fixation in 2% glutaraldehyde (final concentration) dissolved in 50 mM cacodylate buffer (pH 7.4, 150 mM NaCl). This was followed by postfixation in 2% osmium tetroxide (OsO_4_) aqueous solution at a 1:1 volumetric proportion. Subsequent processing involved dehydration through a graded ethanol series and transition to propylene oxide. Specimens were then infiltrated overnight in a 1:1 mixture of propylene oxide and Epon resin. Ultrathin sections (60–80 nm) were generated using an LKB-III ultramicrotome, stained with uranyl acetate (2 h, room temperature), and counterstained with lead citrate (10 min). Final imaging was performed using a JEOL JEM-1200 EX transmission electron microscope.

### 2.7. Fibrin Clot Analysis

Citrated plasma aliquots (100 *μ*L), underwent 15-min preincubation at 37°C with either (a) 1-*μ*L distilled water containing graded recombinant human BDNF (rHu-BDNF) qualities (0.1, 0.2, or 0.3 ng, Solarbio P00062) or (b) equivalent-volume distilled water (control). Following incubation, diluted 1:2 in imidazole buffer, coagulation was triggered by adding CaCl_2_ (2.5 mM final concentration) and human thrombin (0.1 U/mL final concentration, Changchun Lei Yun Shang Pharmaceutical). Polymerization proceeded for 2 h at 37°C, and then, the images were acquired using an optical microscope (Eclipse E200, Nikon, Japan) or laser scanning confocal microscope (Eclipse Ti, Nikon, Tokyo, Japan). Fiber density quantification was performed by blinded investigators using ImageJ software.

### 2.8. Fibrin Polymerization Assay

One hundred microliters of citrated plasma samples was preincubated for 15 min at 37°C with 1 *μ*L of distilled water containing graded rHu-BDNF qualities (0.1, 0.2, or 0.3 ng) or with an equal volume of distilled water (control), diluted 1:2 in imidazole buffer. Plasma clotting was initiated by thrombin (0.1 U/mL) and CaCl_2_ (2.5 mM) (final concentrations). Fibrin polymerization was assessed by a TECAN spectrophotometer at 350 nm with an interval of 30 s.

### 2.9. Rotational Thromboelastography

Plasma coagulation kinetics were assessed using rotational thromboelastometry (ROTEM). Following standardized protocols, 300-*μ*L aliquots of plasma per test condition underwent recalcification with 20-*μ*L CaCl_2_/HEPES buffer followed by activation with thrombin (1 U/mL). Continuous recording commenced immediately upon activation and continued for 60 min per manufacturer specifications. Clot viscoelastic properties were characterized by maximum amplitude (MA), representing peak clot firmness.

### 2.10. Clotting Time

Thrombin clotting time (TT) assessments employed citrated plasma aliquots (100 *μ*L) preincubated for 15 min at 37°C with equal volumes of either (a) 1 *μ*L of distilled water containing graded rHu-BDNF qualities (0.1, 0.2, or 0.3 ng) or (b) distilled water (control). Following incubation, 100 *μ*L of human thrombin (1 U/mL final concentration, Changchun Lei Yun Shang Pharmaceutical) was introduced. The ACL TOP700 system (Werfen) quantified the duration until stable fibrin clot formation.

### 2.11. Clot Lysis Time (CLT)

CLT assays were performed as described before [24]. Plasma coagulation was initiated by adding thrombin (0.1 U/mL) and CaCl_2_ (2.5 mM), concurrently supplemented with recombinant tissue plasminogen activator (rt-PA, 83 ng/mL, Boehringer Ingelheim, Germany). Fibrinolysis kinetics were monitored spectrophotometrically at 350 nm (TECAN Infinite M200) with 30-s interval measurements. The time required for a 50% reduction in maximal turbidity served as the quantitative endpoint for lysis potential assessment.

### 2.12. Statistical Analysis

Statistical analyses were conducted in GraphPad Prism 8.0. Continuous variable distribution normality was verified via Kolmogorov–Smirnov testing. Parametric data are reported as mean ± standard deviation with between-group comparisons using Student's *t*-test. Nonparametric variables are presented as median (interquartile range) and analyzed with Mann–Whitney *U* tests. Categorical data appear as frequency counts (percentages) with Fisher's exact test assessing differences. Pearson correlation coefficients quantified the BDNF-permeability relationship. The significance threshold for all inferential tests was *p* < 0.05.

## 3. Results

### 3.1. Study Subjects

Seventy-two subjects with AIS (35 with T2DM) were enrolled.  Table 1 details baseline characteristics. Compared to nondiabetic (AIS/non-DM) patients, admission serum glucose of AIS/DM patients increased by about 54% (10.98 ± 5.16 vs. 7.11 ± 1.44 mmol/L, *p* < 0.001) and HbA1c levels increased by about 65% (8.31% ± 1.33% vs. 5.05% ± 0.57%, *p* < 0.001). Meanwhile, stroke or TIA history was more frequent in the AIS/DM patients than in the AIS/non-DM group (57.14% vs. 16.22%, *p* < 0.001), and AIS/DM patients had greater stroke severity than AIS/non-DM patients (NIHSS score, 17.09 ± 8.29 vs. 13.22 ± 6.15, *p* = 0.027). Complication rates were comparable between groups. No significant differences emerged in stroke etiology, thrombus location (*p* > 0.05), or preoperative anticoagulant/antiplatelet use. Laboratory assessments revealed comparable fibrinogen levels across groups.

### 3.2. Decreased Plasma BDNF Level and Increased Thrombofibrin Content in AIS Patients With DM

**Figure 1:**
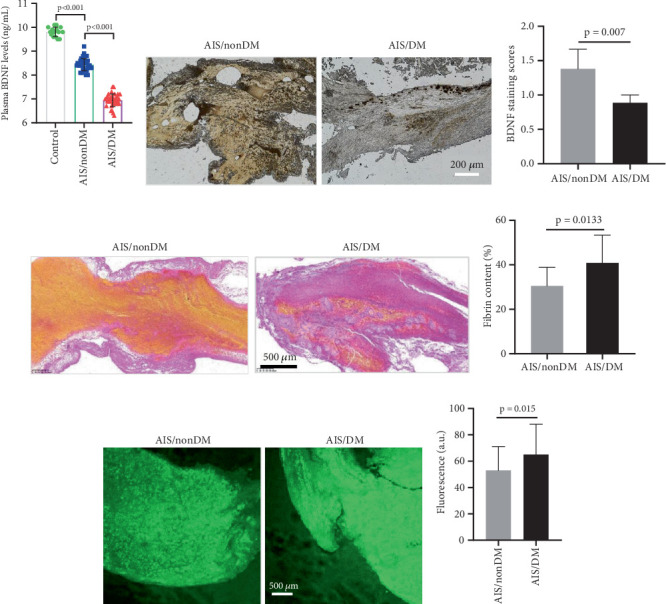
BDNF lower in AIS/DM patients. (a) Expression of plasma BDNF in healthy controls, AIS/non-DM patients, and AIS/DM patients. (b) Representative images of immunohistochemical detection of BDNF (brown) in clots from AIS/non-DM and AIS/DM patients. (c) The column graph represents mean ± SEM of staining scores in each group. (d) Representative clots from AIS/non-DM and AIS/DM patients were stained using MSB to visualize the fibrin (dark pink to red), RBCs (yellow), and platelets (gray). (e) Column graph displays the differences in fibrin content in clots. (f) Fibrin fluorescence of clots from AIS patients. (g) The bar graph represents mean ± SEM fluorescence values.

We measured levels of BDNF in plasma of 72 AIS patients and of 10 healthy subjects (control). In the nondiabetic patients with AIS, BDNF levels were significantly lower than in the control group (*p* < 0.05). Similarly, in the AIS/DM group, BDNF plasma levels were significantly more reduced at admission, in comparison with both controls and nondiabetic patients with AIS (AIS/DM: 6.83 ± 0.81 ng/mL vs. AIS/non-DM: 8.70 ± 0.73 ng/mL, *p* < 0.001, respectively). To determine if BDNF affected the thrombus formed in vivo, we performed immunohistochemical analysis of artery thrombi from MT using a specific antibody for BDNF. As expected, BDNF was detected in thrombi from both AIS/DM and AIS/non-DM patients. The staining intensity, however, was significantly lower (*p* = 0.007, Figure 1b,c) in thrombi from AIS/DM patients, suggesting that BDNF is related to the thrombus formation in vivo. The MSB staining of clots showed that the fibrin content from diabetic patients was higher than that from nondiabetic patients (Figure 1d,e). Immunofluorescence staining analysis showed that the content and density of fibrin from clots in diabetic patients were significantly higher than those in nondiabetic patients (Figure 1f,g).

### 3.3. Positive Correlation Between BDNF and Clot Permeability

**Figure 2:**
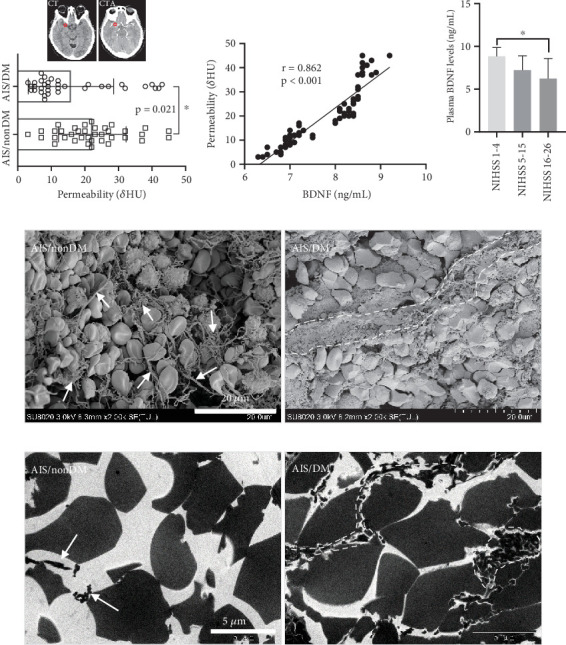
The clot permeability and ultrastructure. (a) Thrombus permeability was significantly lower in AIS/DM patients than AIS patients without DM. (b) Correlation between BDNF concentrations and thrombus permeability. (c) BDNF levels in patients based on the severity of stroke. (d) Scanning electron micrographs of thrombi. Arrows indicate fibrin; dotted lines indicate fibrin band. Bar = 20 *μ*m. (e) Transmission electron micrographs of thrombi from the AIS/non-DM and AIS/DM patients. Arrows indicate fibrin; dotted lines indicate fibrin band. Bar = 5 *μ*m.

To evaluate the association between BDNF levels and thrombus permeability within a clinical cohort, we compared thrombus permeability–based CT and CTA. Interestingly, the permeability of thrombus is higher in the nondiabetic group than in the diabetic group (*p* = 0.021, Figure 2a). In addition, in the entire AIS cohort, a positive correlation between BDNF and thrombus permeability (*r* = 0.862, *p* < 0.001) was found (Figure 2b). A decline in BDNF levels was observed in accordance with the severity of AIS with the least level being in severe stroke (NIHSS ≥ 16, Figure 2c). In order to identify the mechanism responsible for decreased thrombus permeability, we characterized the ultrastructure of clots in vivo. SEM showed that fibrin was hemp rope–shaped in patients with non-DM, and most of the red blood cells in the clot were heterogeneous and not compacted (Figure 2d, left). In contrast, fibrin in the clot of AIS/DM is clustered into a band, and red blood cells are closely packed in the central part of the clot in a polyhedral shape (Figure 2d, right). Furthermore, the TEM also clearly showed the profound differences between the two groups (Figure 2e). On the cross section, the gap between red blood cells in the AIS/non-DM group was large, and fibrin was rare (Figure 2e, left). In the AIS/DM group, however, the gaps between red blood cells are small, and the gaps are filled with fibrin (Figure 2e, right). These data indicate that the difference in thrombus permeability may be partly mediated by BDNF-mediated fibrin changing the microstructure characteristics.

### 3.4. BDNF Inversely Correlates With AIS Patients' Plasma Fibrin Fiber Density In Vitro

**Figure 3:**
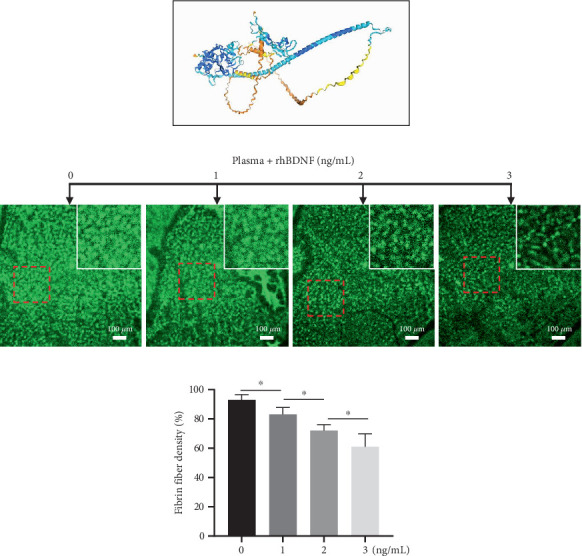
BDNF influences fibrin density. (a) A ribbon diagram illustrates the binding of BDNF to the heparin-binding domain of fibrin(ogen), with the structure generated via DMFold. (b) rHu-BDNF was added to plasma pools from AIS/DM patients before induction of coagulation with thrombin; then, the fibrin density of the clot was analyzed. Visualization images of clots using rabbit polyclonal fibrinogen/FITC (bar = 100 *μ*m) and (c) the percentage of fibrin fibers versus control. Fibrin fibers were analyzed using ImageJ software. All samples were performed in triplicate. Data are expressed as mean ± SEM, ⁣^∗^*p* < 0.05.

Furthermore, given that fibrin(ogen) interacts with BDNF via its heparin-binding domain (Figure 3a) and that the fibrin matrix sequesters BDNF within the clot [25], we proposed that BDNF might alter the structure of fibrin fibers and influence clot stability. Notably, supplementing plasma samples from AIS patients with a baseline BDNF level of 7.0 ng/mL with rHu-BDNF led to a significant decrease in fibrin clot density (Figure 3b,c). Specifically, when the BDNF concentration reached approximately 2 ng/mL, a reduction of roughly 25% in fibrin fiber density was observed (*p* < 0.01).

### 3.5. BDNF Affects the Microstructure of Fibrin

**Figure 4:**
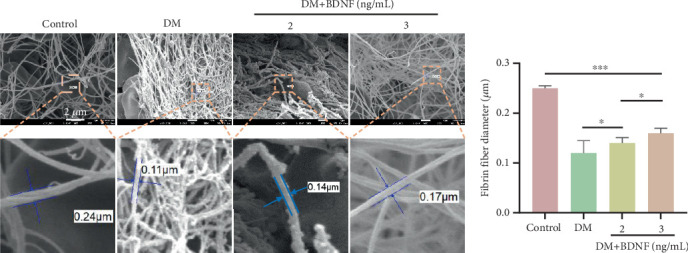
Observing the fibrin diameter by scanning electron microscopy. (a) Fibrin structures formed from venous blood from AIS/DM subjects were added with different concentrations of BDNF. The ultrastructure of fibrin was observed by a scanning electron microscope. (b) The column chart shows the difference in the diameter of fibrin fibers in clots of different groups. ⁣^∗^*p* < 0.05 and ⁣^∗∗∗^*p* < 0.001.

To evaluate BDNF's capacity to modulate fibrin architecture under physiological conditions, rHu-BDNF was introduced into plasma aliquots from AIS/DM subjects (baseline BDNF: 7.0 ng/mL) prior to thrombin-triggered coagulation. SEM showed that the diameter of fibrin in the diabetic group was thin and dense (Figure 4a). Notably, with the increase of BDNF concentration, the fibrin diameter of the clot became thicker and the density decreased (Figure 4b).

### 3.6. Relationship Between BDNF and Fibrin Content in Clots

**Figure 5:**
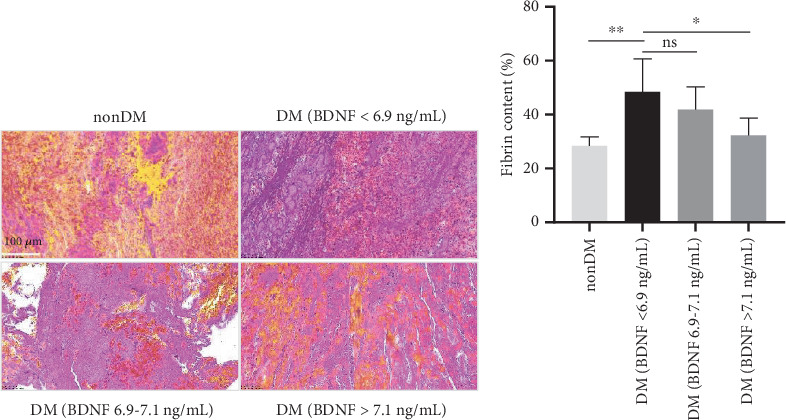
Analysis of fibrin content in blood clot by MSB staining. (a) Representative thrombi from patients with non-DM and DM underwent MSB histochemical staining, to visualize the fibrin (dark pink to red), erythrocytes (yellow), WBCs (blue), and platelets (gray). Scale bar (MSB) = 100 *μ*m. (b) The column chart shows the difference in fibrin content in thrombus among different groups. ⁣^∗^*p* < 0.05 and ⁣^∗∗^*p* < 0.01.

Assessment of the thrombus using MSB staining revealed heterogeneous fibrin components of thrombus in the patient cohort. Compared with those in patients with non-DM, thrombi in AIS/DM patients (BDNF < 6.9 ng/mL) had more fibrin (48.4% vs. 28.4%, respectively, *p* = 0.0023) and fewer RBCs (21.5% vs. 58.2%, respectively, *p* = 0.033) (Figure 5a). In AIS/DM patients, with the high plasma concentration of BDNF, the content of fibrin in blood clots is less (Figure 5a,b).

### 3.7. The Effect of BDNF on Coagulation and Lysis of Clots

**Figure 6:**
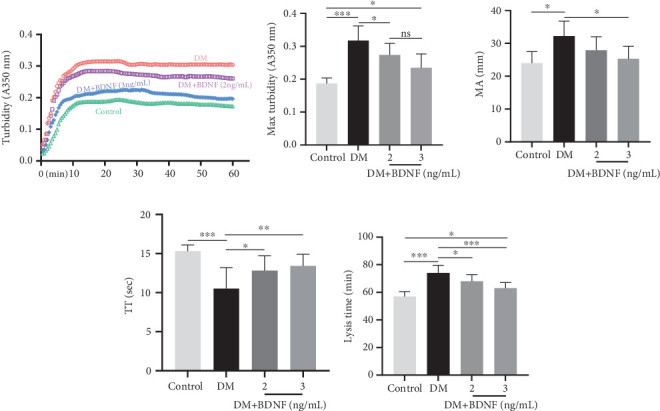
rHu-BDNF influences fibrin polymerization, coagulation, and lysis. (a) Representative turbidity curves were recorded spectrophotometrically at 350 nm (37°C) with 30-s intervals. (b) Maximum turbidity values were determined under identical monitoring conditions. (c) Maximum clot firmness was assessed by thromboelastographic analyses (MA). Plasma samples supplemented with recombinant BDNF prior to thrombin/tPA-induced coagulation underwent subsequent polymerization analysis. (d) Thrombin clotting time (TT) and (e) lysis time were measured. All samples were performed in triplicate. Data are expressed as mean ± SEM, *n* = 3 different pools. ⁣^∗^*p* < 0.05, ⁣^∗∗^*p* < 0.01, and ⁣^∗∗∗^*p* < 0.001.

Prior to thrombin-induced coagulation, plasma aliquots from AIS/DM subjects (baseline BDNF: 7.0 ng/mL) were supplemented with rHu-BDNF. Subsequent analyses assessed clot polymerization kinetics and viscoelastic properties. Polymerization of clots was assessed by turbidity assay, and viscoelastic properties were analyzed by rotational thromboelastography. The increase of rHu-BDNF concentration reduces the polymerization rate (Figure 6a), and the maximum turbidity also decreases (Figure 6b). Additional addition of rHu-BDNF (2 or 3 ng/mL) reduced the firmness of the clot as shown by the reduction in MA (Figure 6c). Interestingly, both concentrations of rHu-BDNF prolonged TT (Figure 6d). And rHu-BDNF affected significantly the lysis time (Figure 6e).

## 4. Discussion

The present study provides novel insights into the role of BDNF in modulating fibrin-rich thrombus formation in T2DM patients with AIS. Our key findings demonstrate that (1) plasma BDNF levels are significantly reduced in AIS patients with T2DM compared to nondiabetic AIS patients and healthy controls; (2) reduced BDNF correlates with higher fibrin density, lower thrombus permeability, and increased stroke severity (NIHSS); (3) in vitro supplementation of rHu-BDNF restores physiological clot morphology by reducing fibrin density, prolonging clotting time, and enhancing fibrinolysis. These results collectively support our initial hypothesis that BDNF deficiency contributes to pathological thrombus formation in diabetic stroke, partially through altered fibrin ultrastructure.

Our observation of decreased BDNF in AIS/DM patients aligns with prior clinical studies reporting reduced BDNF in diabetic populations [9–11], potentially due to platelet BDNF depletion from hyperglycemia-induced platelet hyperactivity [12]. However, conflicting reports exist—some studies associate T2DM with elevated BDNF [26, 27], possibly linked to metformin treatment [28]. In the present study, 83% (29/35) of diabetic patients were on metformin therapy. Despite this, BDNF levels remained significantly lower in our AIS/DM cohort compared to nondiabetic AIS patients. This discrepancy may be attributed to several factors. Firstly, the duration and dosage of metformin treatment in our population might differ from those in studies reporting elevated BDNF. Secondly, the specific patient characteristics, such as poorer glycemic control (mean HbA1c of 8.31% in our cohort), more severe hyperglycemia, or the co-occurrence of AIS, might counteract the potential BDNF-elevating effects of metformin. Finally, differences in sample processing or BDNF measurement assays could also contribute to the observed variations. Therefore, our findings suggest that in the context of AIS complicated with diabetes, the disease-specific factors may override the potential BDNF-modulating effects of metformin.

Crucially, our study extends current understanding by establishing a direct link between BDNF and fibrin architecture in the context of diabetic stroke. Through histopathological and electron microscopy analyses, we demonstrate that BDNF deficiency is associated with the formation of dense, fibrin-rich thrombi with reduced permeability—a phenotype resistant to thrombolysis [15]. These findings align with Amadio et al. [18], who proposed low levels of BDNF are associated with the formation of denser, larger clots in vitro and that exogenous BDNF could reduce fibrin fiber density and clot firmness.

Notably, our results further reveal that BDNF not only reduces fibrin density (Figures 3 and 4) but also enhances clot lysis (Figure 6e), supporting a protective role against pathological clotting. The apparent consistency in fibrin-modifying effects between the two studies—both using human samples—suggests a conserved mechanism, though contextual differences such as patient population (AIS with T2DM vs. CHD) and methodological approaches may contribute to nuanced mechanistic interpretations.

Moreover, our in vitro functional assays provide deeper mechanistic insight into how BDNF influences clot architecture and stability. The addition of rHu-BDNF to AIS plasma dose-dependently attenuated fibrin polymerization, reduced fibrin fiber density, increased fiber diameter, decreased clot firmness and turbidity, prolonged clotting time, and accelerated clot lysis (Figure 6). These effects are consistent with BDNF's interaction with the heparin-binding domain of fibrin(ogen) (Figure 3a) [24], which likely alters fibrin polymerization kinetics and fiber ultrastructure, leading to the formation of looser, more lytic networks with modified viscoelastic properties. The strong positive correlation between BDNF levels and thrombus permeability (Figure 2b) further underscores the clinical relevance of BDNF in thrombus composition and remodeling.

Furthermore, the striking presence of polyhedral erythrocytes (polyhedrocytes) within the core of thrombi from AIS/DM patients (Figure 2d,e) provides critical morphological insight into the pathophysiology of these clots. The formation of polyhedrocytes is a well-established hallmark of intense intravital clot contraction, a process driven by platelet activation and contraction that propagates through the fibrin network [29]. Our observation of polyhedrocytes in diabetic thrombi suggests that a highly contractile and stable thrombus has formed in vivo. Although our in vitro studies focused on the direct effects of BDNF on fibrin structure in platelet-poor plasma, the in vivo reality involves complex interactions within whole blood. We suppose that the hyperactive platelet state coupled with reduced BDNF levels in AIS/DM patients promotes fibrin polymerization and increased fibrin rigidity, ultimately leading to the formation of these compacted, fibrin-rich thrombi containing polyhedrocytes.

Moreover, the robust positive correlation we observed between plasma BDNF levels and thrombus permeability—assessed both radiologically (via CT/CTA) and ultrastructurally (via electron microscopy)—was further supported by functional in vitro assays demonstrating that BDNF enhances fibrinolytic susceptibility and reduces clot density. This consistency across imaging, morphological, and biochemical evaluations strengthens the conclusion that BDNF contributes to a more permeable and lytic thrombus phenotype.

Nonetheless, our study has limitations. First, the sample size was modest, and subgroup analyses, particularly for clot composition and functional assays, may be underpowered. Second, while we provide compelling associative and mechanistic data, causality between BDNF depletion and impaired thrombus resolution cannot be conclusively established without interventional in vivo studies. Third, BDNF levels may be influenced by confounding factors such as medication (e.g., metformin), stress response, or platelet reactivity, which were not fully controlled for. Lastly, we did not explore the potential involvement of BDNF receptors (e.g., TrkB) or downstream signaling pathways in clot regulation, which warrants further investigation.

## 5. Conclusion

In conclusion, our study reveals that reduced BDNF levels in AIS patients with T2DM are associated with the formation of denser, less permeable, fibrin-rich thrombi, which may contribute to thrombolysis resistance and worse stroke outcomes. BDNF modulates fibrin structure and clot stability in a concentration-dependent manner. Collectively, these findings position BDNF as both a biomarker of thrombotic risk and a potential therapeutic target in diabetic stroke. Future research should validate these results in larger cohorts, explore BDNF supplementation strategies in diabetic stroke models, and investigate the clinical utility of BDNF-based interventions within thrombolysis protocols.

## Figures and Tables

**Table 1:** AIS patients' clinical characteristics at baseline.

**Variable** **N** = 72	**Non-DM** **n** = 37	**DM** **n** = 35	**p**
Male (*N*)	18 (48.6%)	20 (57.1%)	0.489
Age (years)	71.03 ± 13.5	71.23 ± 11.66	0.952
Stroke or TIA history	6 (16.22%)	20 (57.14%)	< 0.001
Atrial fibrillation	11 (29.73%)	18 (51.43%)	0.092
Coronary artery disease	11 (29.73%)	19 (54.29%)	0.055
Dyslipidemia	17 (45.95%)	24 (68.57%)	0.061
Hypertension	20 (54.05%)	26 (74.29%)	0.089
Glycemia (mmol/L)	7.11 ± 1.44	10.98 ± 5.16	< 0.001
HbA1c (%)	5.05 ± 0.57	8.31 ± 1.33	< 0.001
Smoking history	10 (27.03%)	17 (48.57%)	0.088
Fibrinogen (g/L)	3.24 ± 0.58	3.21 ± 0.66	0.873
NIHSS	13.22 ± 6.15	17.09 ± 8.29	0.027
Anticoagulant use	10 (27.03%)	14 (40%)	0.318
Antiplatelet use	19 (51.35%)	16 (45.71%)	0.646
Stroke etiology			0.287
Cardiogenic embolism	24	15	
Large artery atherosclerosis	8	14	
Other determined	1	1	
Cryptogenic	4	5	
Thrombus location			0.537
ICA	10	14	
M1	13	9	
M2	5	3	
ACA	1	3	
BA	8	6	

*Note:* Results are presented as median (IQR), number (percentage), or mean ± SD.

Abbreviations: ACA, anterior cerebral artery; BA, basilar artery; ICA, internal carotid artery; NIHSS, National Institutes of Health Stroke Scale; TIA, transient ischemic attack.

## Data Availability

Data from the manuscript can be available on reasonable request.
